# Topical Hyperbaric Oxygen Therapy Versus Local Ozone Therapy in Healing of Venous Leg Ulcers

**DOI:** 10.3390/ijerph20031967

**Published:** 2023-01-20

**Authors:** Jarosław Pasek, Sebastian Szajkowski, Valter Travagli, Grzegorz Cieślar

**Affiliations:** 1 Faculty of Health Sciences, Jan Długosz University in Częstochowa, 13/15 Armii Krajowej St., 42-200 Częstochowa, Poland; 2 Faculty of Medical Sciences, Medical University of Mazovia in Warsaw, 8 Rydygiera St., 01-793 Warszawa, Poland; 3 Department of Biotechnology, Chemistry and Pharmacy, Viale Aldo Moro 2, 53100 Siena, Italy; 4 Department of Internal Medicine, Angiology and Physical Medicine, Faculty of Medical Sciences in Zabrze, Medical University of Silesia in Katowice, 15 Stefana Batorego St., 41-902 Bytom, Poland

**Keywords:** chronic wounds, venous leg ulcers, hyperbaric oxygen therapy, local ozone therapy, ulcer surface area, pain intensity

## Abstract

Background: the treatment of venous leg ulcers still poses a difficult interdisciplinary medical problem. The aim of this study was to compare the therapeutic efficacy of local hyperbaric oxygen therapy with local ozone therapy in the treatment of venous leg ulcers. Materials: this study included 114 patients; 60 males (52.63%) and 54 females (47.36%) of ages ranging between 39 and 88 years (mean age: 68.9 ± 9.8 years) with venous leg ulcers, who underwent topical hyperbaric oxygen therapy (group I) and local ozone therapy (group II). In each of the study groups, the patients underwent 30 therapeutic procedures lasting 30 min each. The progress in wound healing was evaluated by computerized planimetry, and the intensity of pain was assessed with the use of the Visual Analogue Scale (VAS). Results: in both groups of treated patients, a statistically significant (*p* = 0.000001) reduction in the area of treated ulcers was achieved. In group I, the wound area decreased by an average of 69.67 ± 22.52%, from 7.55 ± 2.99 cm^2^ to 2.78 ± 2.43 cm^2^, and in group II, by an average of 41.33 ± 21.31%, from 7.36 ± 2.82 cm^2^ to 4.62 ± 2.76 cm^2^. In both groups of patients, a statistically significant (*p* = 0.000001) reduction in the intensity of pain ailments was observed: in group I, by an average of 0.55 ± 0.54 points, and in group II, by an average of 2.33 ± 0.82 points on the VAS scale. Conclusions: local hyperbaric oxygen therapy and local ozone therapy cause a statistically significant reduction in the surface area of venous leg ulcers as well as in the intensity of pain. Better results were observed after the application of local hyperbaric oxygen therapy procedures.

## 1. Introduction

As perceived worldwide, the problem of wounds that are difficult to heal affects approximately 20 million patients [1]. In Poland, according to current statistical data, difficult-to-heal wounds occur in about 760 thousand patients [2]. The most common chronic wounds are ulcers occurring in the course of diabetic foot syndrome, venous ulcers, and arterial ulcers in the course of obstructive artery disease of the lower extremities. Other causes of chronic wounds include pressure ulcers, burns, connective tissue diseases, injuries, and cancer. Statistics show that the number of patients with this problem will increase due to the ever more common occurrence of civilization diseases [2,3].

The modern concept of treatment of chronic wounds assumes its full complexity. The treatment should primarily target the underlying disease, with the aim of, inter alia, elimination of risk factors responsible for abnormal healing, e.g., obesity, diabetes, malnutrition, atherosclerosis, or nicotinism [3,4,5,6,7,8]. Local wound treatment, in turn, should take into account wound management according to the currently developed TIMERS strategy (T—tissue management, I—infection and inflammation control, M—moisture balance, E—epithelial advancement, R—repair and regeneration, and S—social- and individual-related factors) sequentially comprising cleaning of the wound, controlling infection and inflammation in the wound, ensuring adequate moisture level, stimulating epithelialization, regeneration of tissue, and taking social factors into account. According to experts, only such an approach can ensure the expected results of treatment [9].

Venous leg ulcers occur in about 1–3% of the general population [10]. These ulcers are consequences of pathological changes occurring in venous vessels of various etiologies. The most common causes include chronic venous insufficiency, varicose veins of lower limbs, and deep vein thrombosis. In the age group > 65 years of age, their incidence is at almost 5% [11]. In the case of about 70% of patients, ulcers recur within 3 months after their healing [4,10,12].

Physical treatment has been gaining popularity and is ever more widely used in various fields of medicine. For several years, an increasing number of studies have been carried out to determine the effectiveness of physical medicine; such studies included hyperbaric oxygen therapy and ozone therapy in the treatment of chronic and difficult-to-heal wounds [13,14,15].

Hyperbaric oxygen therapy (HBO) procedures have been used for many years in medicine in the treatment of difficult-to-heal wounds of various etiologies. In the case of topical hyperbaric oxygen therapy, the treated limb (upper or lower one) is placed in a cylindrical therapeutic chamber which is sealed with a polyethylene collar. Then, 100% oxygen with a pressure higher than 1 ATA is pumped into the chamber (flow rate of about 10 L/min), which directly affects the wound being treated [13,16].

Both topical normobaric oxygenation and topical HBO procedures are effective (in terms of healing efficiency and the frequency of relapses), in similarity to conventional systemic hyperbaric oxygen therapy conducted in hyperbaric chambers, but do not cause systemic complications. Therefore, topical HBO therapy could be an alternative method, especially for patients with numerous contraindications that could disqualify such patients from using the procedures in systemic hyperbaric chambers [13,17,18,19,20].

Locally increased tissue oxygenation occurring in the case of the use of HBO results in, among others, the acceleration of fibroblast proliferation, faster regeneration of ischemic parts of the skin, acceleration of the process of granulation tissue growth and wound epithelialization, intensification of the angiogenesis process and improvement in the supply of arterial blood to tissues, as well as drainage from venous blood tissues [21]. Topical HBO is a simple method that does not require a special room for its application, and the oxygen for the treatment procedures can be administered from a typical oxygen cylinder intended for medical purposes [13,16,22].

The use of HBO allows the acceleration of ulcer healing processes. Hypoxia (pO_2_ = 0–15 mmHg) occurs in tissues surrounding the wound, due to impaired microcirculation and increased metabolism. This causes an increase in oxygen consumption around the periphery of the wound and its reduced supply to damaged tissues. The gradient of oxygen concentration is eliminated, which leads to the inhibition of new granulation tissue formation because of the angiogenesis-stimulating factor. In hypoxia, fibroblasts are unable to produce collagen. Only the supply of O_2_ in the right concentration allows the production and deposition of collagen in the tissues to start. Neovascularization requires the participation of both endothelial cells and fibroblasts to produce collagen. Both factors need oxygen to function properly. Oxygen concentrations higher than physiological ones also accelerate epidermalization [23,24].

Ozone is an active form of oxygen used in the form of an oxygen–ozone mixture (up to 5% of ozone and at least 95% of oxygen). Such treatment is called “Ozone bags” or ”Ozone shoes”. In the case of local treatment, the ozone concentration should be in the range of 30–100 μg /mL of oxygen. In devices generating ozone from medical oxygen, the ozone concentration ranges from 5 to 70 μg/mL, and its pressure should be 0.06 Mpa [14]. One of the therapeutic mechanisms of topical ozone therapy, especially in patients with infected wounds, is the bactericidal effect related to the destruction of bacterial cell membranes due to the oxidation of polyunsaturated fatty acids forming those membranes, and enzymatic proteins in the cytoplasm by active singlet oxygen (acting as a strong oxidant generated during the dissociation of ozone molecule), resulting in disturbances of the activity of numerous cellular organelles, damage to DNA and, consequently, in bacterial cell apoptosis [25,26]. Ozone also reduces inflammation in the vicinity of the wound by inhibiting the migration of mast cells, reducing the release of lysosomal enzymes and some acute phase proteins, inhibiting the formation of eosinophilic infiltration, and stimulating the synthesis of antioxidants [27,28]. Moreover, red blood cells (erythrocytes) become more flexible under the influence of ozone, which results in the increase in tissue oxygenation and nutrient supply and, consequently, in the intensification of the healing process [27,29].

Clinical studies show that ozone improves local tissue blood supply, stimulates granulation and epithelization, and accelerates ulcer healing. The analgesic effect of ozone therapy is also significant. The authors claim that the obtained effect is the result of activation of the expression of antioxidant factors, VEGF, PDGF, and TGF-β. The studies also showed a reduced tissue infiltration with inflammatory cells, and a reduction in the expression of pro-inflammatory factors. Stimulation of angiogenesis, collagen production, and keratinization of the stratum corneum were also observed [30,31,32].

The conservative methods used in the comprehensive treatment of venous leg ulcers include pharmacotherapy, specialist dressings, VAC negative pressure therapy, proper diet, regular physical activity, and selected physical medicine treatment procedures, including hyperbaric oxygen therapy and ozone therapy. Systemic use of hyperbaric oxygen in hyperbaric chambers is subject to numerous contraindications that may disqualify patients from eligibility to this form of treatment. In our study, we used the local application of both physical agents, which is characterized by a negligible number of contraindications and side effects. It should be added that the local application of both therapeutic agents is non-invasive, painless, and not burdensome, which is very important for the patients treated. By improving the effectiveness and shortening the course of the treatment process, these procedures reduce the number of surgical procedures (limb amputations), which translates into an improvement in the quality of life, and which enables patients to live independently [15,33,34].

## 2. Aim of the Study

The aim of this study was to compare the therapeutic efficacy of local hyperbaric oxygen therapy versus local ozone therapy in the treatment of venous leg ulcers.

## 3. Material and Methods

The Ethics Committee of the University of Silesia in Katowice granted ethical approval (permission No.: KNW/0022/KB1/102/16), Poland. All qualified patients signed a written informed consent for participation in this study.

This study included all 114 patients (60 men and 54 women), aged 39 to 88 years (68.9 ± 9.8 years), hospitalized at the Department of Internal Medicine, Angiology, and Physical Medicine in Bytom in the period between 2017 and 2021 with diagnosis of venous ulcers located on the right or left lower limb, who gave their consent for participation in this study. The disease duration was from 1 to 9 years (mean period: 4.85 ± 1.56 years). The patients were enrolled in two research groups, alternately, taking into account the contraindications of both methods of therapy. The first research group consisted of 60 patients (25 women and 35 men) undergoing local hyperbaric oxygen therapy using the OXYBARIA-S device by FASER SA (Ruda Śląska, Poland) [22]. The second research group consisted of 54 patients (29 women and 25 men) who underwent local ozone therapy using the Ato-3 device (Metrum Cryoflex, Blizne Łaszczyńskiego, Poland) [14].

The mean age of patients in group I (70.0 ± 7.9 years) did not differ statistically significantly when compared to those in group II (66.7 ± 11.9 years) (*p* = 0.138). The Body Mass Index (BMI) values in group I were 24.3 ± 1.83 kg/m^2^ on average, while in group II those values amounted to 25.3 ± 2.83 kg/m^2^—they did not differ statistically significantly (*p* = 0.067). Similarly, the mean disease duration in group in the range of 4.76 ± 1.58 years did not differ statistically significantly from the mean disease duration in group II, which was in the range of 5.01 ± 1.54 years (*p* = 0.465).

The inclusion criteria were as follows: Presence of leg ulcer located in the area of the right or left lower limb, resulting from venous insufficiency, patient’s age over 18 years, patient’s consent to participate in this study.

The exclusion criteria were as follows: Ulcer etiology other than venous one, local infection of the ulcer requiring antibiotic therapy, chronic ischemia of lower limbs—ABI value of less than 0.8, active neoplastic disease, deep vein thrombosis, absence of the patient’s consent to participate in this study, standard contraindications to the application of the physical methods analyzed.

Before commencing with physical procedures, each patient underwent a Doppler scan to determine the etiology of the ulcer. Each patient also underwent a surgical and angiological consultation. Surgical wound debridement was performed in case of the presence of necrotic tissue or purulent infiltration.

### 3.1. The Physical Treatment Procedure

Patients from group I underwent a series of topical hyperbaric oxygen therapy treatment procedures with the use of the OXYBARIA-S apparatus. During the procedures, oxygen at a concentration of 95%, with a pressure of 1.5 Ba (1.485 ATA) and flow rate of about 5 L/min, was introduced from an external cylinder into a cylindrical therapeutic chamber in which the limb with ulceration was placed, and sealed on the surface of thigh of the treated limb by means of a flexible sealing collar, where it affected the ulcer surface directly.

Patients from group II underwent a series of topical ozone therapy treatment procedures with the use of the Ato-3 device. During the procedures, the oxygen–ozone mixture (5% of ozone and 95% of oxygen) with a concentration of 40 μg/mL, generated in the device, was inserted into a flexible plastic bag (the so-called ozone shoe) in which the limb with ulceration was placed, sealed on the surface of the treated thigh limbs by means of an elastic latex band, where it affected the ulcer surface directly.

In both study groups, treatment procedures were performed every day (except Saturdays and Sundays) in two series of 15 treatment procedures, and the duration of a single treatment was 30 min. The interval between consecutive series was 4 weeks, during which, according to our clinical experience (unpublished data), there was a sustained self-regeneration of the tissues surrounding the ulcers induced by physical factors. The physical treatment procedures conducted as part of this study were performed by one specialist with experience in the field of physical medicine.

After each treatment procedure, the ulcers were treated with antiallergic adhesive foam silver dressings (Allevyn adhesive Ag, Berkshire, UK) in order to ensure antiseptic conditions and mechanical protection of the ulcer. All patients were treated with local compression therapy with the use of elastic bandage (Codoban, Tricomed, Łódź, Poland) and local pharmacological treatment, including sulodexide, micronized purified flavonoid fraction, pentoxifylline, and acetylsalicylic acid in standard doses.

High-resolution digital photographs were used to assess ulcerations in this study. Original computer software for planimetric assessment of the ulceration surface area was used for digital image processing. The program automatically calculated the size of the wound surface area and, after the scaling process, gave the result in square centimeters. In order to maintain high accuracy, all measurements were performed by one specialist experienced in this method who did not know what physical method of examination was applied [15].

Moreover, before the beginning and after the end of the therapeutic cycle, pain intensity was assessed in both groups using a 10-point VAS scale.

### 3.2. Statistical Analysis

The statistical analysis of the collected data was performed using Statistica 13 software package (Statsoft, Kraków, Poland). The Shapiro–Wilk test was used to test the normality of data. There were some non-normal distributions of data. The results are presented as mean values and standard deviation. The Wilcoxon test was used to test the statistical significance of the differences in the examined parameters before and after the treatment was applied. The statistical significance of differences between the groups was tested using the Mann–Whitney U test. The level of statistical significance was set at *p* < 0.05.

## 4. Results

**Figure 1:**
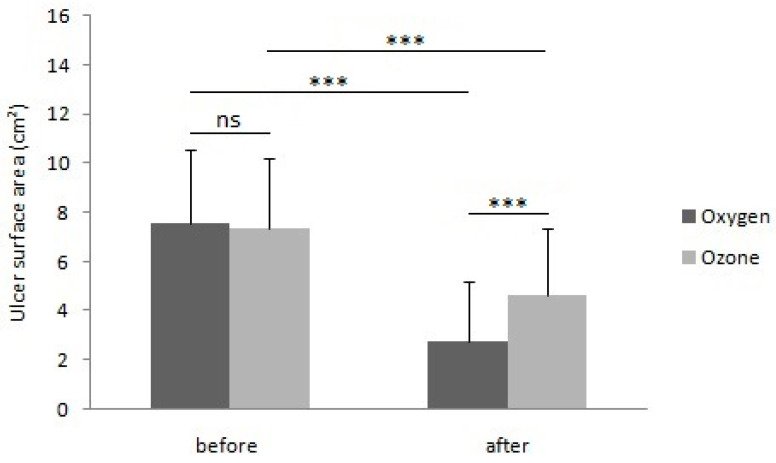
Comparison of results of surface area measurements for venous leg ulcers in patients treated with local hyperbaric oxygen therapy and local ozone therapy, before and after the treatment cycle. (ns: Not significant; *** *p* < 0.001).

**Figure 2:**
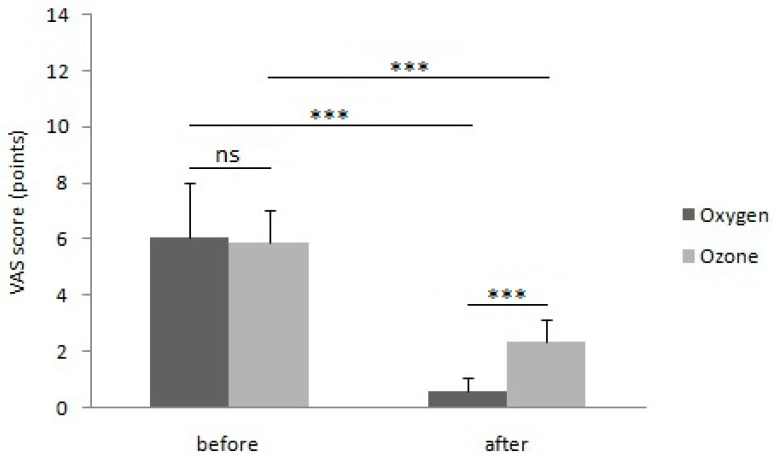
Comparison of pain intensity assessed using the VAS scale in patients treated by means of local hyperbaric oxygen therapy and ozone therapy, before and after the treatment cycle. (ns: not significant; *** *p* < 0.001).

A part of the results regarding patients from group II was previously published [20]. Before the start of physical treatment procedures, the ulcer surface area in group I averaged 7.55 ± 2.99 cm^2^, and it did not differ statistically significantly as compared to the ulcer surface area in group II, which averaged 7.36 ± 2.82 cm^2^ (*p* = 0.675) (Figure 1). Similarly, no statistically significant differences were observed regarding the intensity of pain ailments between group I, where the pain intensity in the VAS scale was 6.05 ± 1.90 points, and group II, where the pain intensity in the VAS scale averaged 5.88 ± 1.17 points (*p* = 0.207) (Figure 2).

After the completion of treatment, the ulcer surface area in group I averaged 2.78 ± 2.43 cm^2^, and it was statistically significantly lower compared to the ulcer area in group II, averaging 4.62 ± 2.76 cm^2^ (*p* < 0.001) there (Figure 1).

Similarly, after completion of the treatment procedure, the intensity of pain assessed on the VAS scale, which averaged 0.55 ± 0.54 points in group I, was statistically significantly lower compared to the intensity of pain in group II, amounting to an average of 2.33 ± 0.82 points (*p* < 0.001) (Figure 2).

The comparison of measurements of the ulcer area before and after the treatment cycle indicated that both study groups showed a statistically significant reduction in the ulcer area, in group I from the average of 7.55 ± 2.99 cm^2^ to the average of 2.78 ± 2.43 cm^2^ (*p* < 0.001), and in group II from the average of 7.36 ± 2.82 cm^2^ to the mean result of 4.62 ± 2.76 cm^2^(*p* < 0.001) (Figure 1). Similarly, by comparing the intensity of pain assessed using the VAS scale before and after the treatment cycle, both research groups showed a statistically significant reduction in pain intensity, in the case of group I from the average of 6.05 ± 1.9 points to 0.55 ± 0.54 points (*p* < 0.001), and in group II from the average of 5.88 ± 1.17 points to the average of 2.33 ± 0.82 points (*p* < 0.001) (Figure 2).

The percentage reduction in the ulcer surface area as a result of the applied physical treatment, which amounted to 69.67 ± 22.52% in group I, was statistically significantly greater than in group II, in which it amounted to 41.33 ± 21.31 (*p* < 0.001). On the other hand, the percentage reduction in pain intensity assessed using the VAS scale, which in group I averaged 88.94 ± 13.64%, was statistically significantly bigger than in group II, in which it amounted to 60 ± 12.97% (*p* < 0.001).

In 46 patients (76.66%) in group I, the reduction in the ulcer surface area above 50% of the baseline values was achieved, while in group II, the reduction in the ulcer surface area above 50% of the baseline values was found in 18 patients (33.33%). Complete ulcer healing was observed in 17 patients (28.33%) in group I and in 2 patients (3.70%) in group II. In none of the patients, both in group I and group II, was any enlargement of the ulcer surface noted after the applied treatment.

In all patients from group I, a reduction in pain intensity exceeding 50% of the initial values was achieved, while in group II the reduction in pain intensity exceeding 50% of the initial values was found in 50 patients (92.59%). Complete pain relief was observed in 28 patients (46.66%) in group I and 2 patients (3.70%) in group II. In none of the patients, both in group I and group II, was an increase in pain after the treatment detected.

All patients treated by means of either physical method used tolerated the procedures well, and no side effects were observed in any of the patients during the course of treatment.

After completing our study, we summarized the costs related to the treatment applied in both study groups. The total cost of the therapy, including local hyperbaric oxygen therapy procedures (EUR 3145), daily dressings with specialist Ag dressing (EUR 9605), Codoban elastic bandages for compression therapy (EUR 680), and other dressing materials (EUR 2890), amounted to some EUR 16,320.

On the other hand, the total cost of the therapy, including ozone therapy procedures (EUR 2960), daily dressings with specialist Ag dressing (EUR 7662), Codoban elastic bandages for compression therapy (EUR 632), and other dressing materials (EUR 2528), amounted to some EUR 13.782. The total summarized costs of both physical therapies appear to be similar.

## 5. Discussion

Modern medical equipment and innovative methods of physical therapy significantly extend the indications for the use of physical treatments and increase the effectiveness of broadly understood therapeutic rehabilitation. Over a dozen or so years, more and more reports have appeared in the literature concerning the beneficial effects of using physical medicine methods, including hyperbaric oxygen therapy and local ozone therapy in the treatment of hard-to-heal wounds, where those methods stimulate the natural regeneration processes of tissues, providing an appropriate environment for the healing process [13,17,35,36,37,38,39].

According to the latest data from the American Committee of Hyperbaric Medicine (ACHM), wound treatment should be conducted in a comprehensive manner, as only a multidirectional treatment model gives the best therapeutic effects [40]. According to Ozler et al., the therapeutic mechanisms of HBO and ozone therapy are similar; therefore, it can be expected that the effect of these two physical factors may induce the desired synergistic effects in the treatment of chronic wounds that are difficult to heal [29].

The results of previous studies concerning the use of HBO in the treatment of chronic leg ulcers of various etiologies, as well as our own study, confirm the therapeutic effectiveness of this method in relation to the reduction in the surface area of treated ulcers. It is related to the percutaneous application of hyperbaric oxygen, which is a condition for the improvement of tissue oxygenation in the ulcer area, which was confirmed, among others, by the study of Pasek et al. [21]. The appropriate amount of oxygen supplied to tissues is necessary for the proper development of successive stages of ulcer healing. Appropriate oxygen partial pressure in the tissues of the ulceration area is also necessary to maintain the required activity of enzymes involved in the processes of infection reduction, collagen synthesis, angiogenesis, and epithelialization.

Poyrazoglu et al. evaluated the effects of HBO and HBO preconditioning (pre-HBO) on experimental wound healing and tensile strength in the colonic anastomosis in rats. In their research, the authors showed that the use of HBO accelerated wound healing and strengthened the tissue in terms of its elasticity. It seems that HBO may aid in wound healing in colon anastomosis [41].

Yildirim et al. confirmed, in an experimental study on an animal model, that HBO and ozone therapy may have positive therapeutic effects on the edema, inflammation, and ischemia occurring due to trauma. Soft tissue trauma was caused in 63 adult Sprague Dawley rats divided into three groups: a first group subjected to HBO therapy procedures for 7 days, a second group subjected to ozone therapy procedures for 7 days, and the control group. In both experimental groups, a significant decrease in inducible nitric oxide synthase and tissue lipid peroxidation levels, as well as a reduction in inflammation and edema in the histopathological assessment, were observed, as compared to control animals [42].

The results of our study regarding the analgesic effect of HBO are consistent with the results of the clinical trial of Lalieu et al., in which the authors, in a retrospective, single-center cohort study, focused on patients with chronic wounds treated in outpatient conditions with HBO therapy. The patients’ quality of life was assessed using the EQ-5D-5L questionnaire, and the existing pain was assessed. The analysis of the results showed an improvement in the quality of life (*p* = 0.02) and a significant reduction in pain intensity (*p* < 0.001) [43].

In another clinical study, Suehiro et al. showed the beneficial therapeutic effects of HBO in five cases of venous leg ulcers (VLU) that were resistant to conservative therapy. All VLUs were healed after 17–66 sessions of HBO therapy [44].

Thistlethwaite et al. confirmed the effectiveness of hyperbaric oxygen therapy on difficult-to-heal venous leg ulcers in 74 patients. A total of 43 patients achieved greater than 50% improvement in their treated ulcers 4 weeks after starting treatment. According to the authors, HBO therapy may improve refractory healing in venous leg ulcers, and HBO therapy may be applied as an adjunct treatment for non-healing patients, returning indolent ulcers to a healing trajectory [45].

In turn, Altinel et al. compared the effects of HBO therapy and ozone therapy in an experimental study in which a model of forty rats with acute distal colitis (ADC) was exposed to those therapeutic methods in order to reduce inflammatory pathological conditions. The results obtained showed that the therapeutic effect of ozone therapy is more pronounced than that of HBO therapy, and its possible mechanisms are related to decreasing inflammation, edema, and oxidative stress [46].

The mechanisms of the therapeutic effect of HBO also include the stimulation of neovascularization processes, which—under elevated pressure—are induced by the stimulation of VEGF synthesis, which leads to increased activity of progenitor cells and the intensification of new vessel formation, as well as stimulation of nitric oxide synthase (NOS) production and, consequently, increased synthesis of nitric oxide (NO). HBO also stimulates the process of angiogenesis by increasing the activity of the basic fibroblast growth factor (bFGF), as well as building an oxygen concentration gradient between the central area of the wound and its margins [29,42,47].

The mechanisms of the therapeutic effect of local ozone therapy include, among others/inter alia, securing the wound against the development of infection by reducing the activity of bacterial cells, as well as improving tissue metabolism by increasing oxygenation and reducing local inflammation. The ozone which is delivered to tissues during the treatment procedures is transformed into singlet oxygen, which causes oxidation of polyunsaturated fatty acid forming bacterial cell membranes and enzymatic proteins in the cell cytoplasm, resulting in the complete destruction of the cell membrane, disturbances of the activity of numerous cellular organelles, injury of DNA and, consequently, in bacterial cell apoptosis [25,26]. At the same time, ultra-short exposure of tissues subjected to ozone therapy prevents the formation of microbial strains resistant to this type of treatment. Moreover, ozone influences blood circulation and improves microcirculation in tissues, and also stimulates angiogenesis, while its analgesic effect is related to local inhibition of inflammation in the area of ulceration and stimulation of the activity of the endogenous opioid system [29].

The results of published clinical trials, in which local hyperbaric oxygen therapy was used in the treatment of chronic leg ulcers of various etiologies, also indicate the therapeutic efficacy of this method, related to, e.g., a reduction in the intensity of the perceived pain, which was also unequivocally confirmed in our study.

Despite intensive clinical and experimental research, there is still no generally accepted, effective method for the treatment of chronic, hard-to-heal leg ulcers. Pharmacological treatment raises some hopes, while alternative methods of therapy for difficult-to-heal wounds include, among others, preparations of platelet-rich plasma and cell growth factors, negative pressure dressings, as well as dressings containing silver ions with antibacterial properties. Intensive development is also noted as regards the wide use of physical methods, which include, among others, HBO and ozone therapy [39].

In our study, we have shown that the use of local hyperbaric oxygen therapy in the treatment of patients with chronic venous leg ulcers resulted in more favorable therapeutic effects in terms of stimulation of the healing process and pain relief, as compared to ozone therapy. Bearing this in mind, it should be emphasized that when planning comprehensive treatment of difficult-to-heal wounds, including leg ulcers, apart from proper wound care and appropriate pharmacotherapy, the selection of optimal physical treatment methods should also be taken into account.

It is estimated that in Europe, the costs of treatment and care for patients with chronic wounds account for up to 3–5% of the budgets of European healthcare systems. Modern medicine is constantly looking for alternative methods of effective treatment of chronic wounds, with regard to their costs. As was presented in the Results chapter, the summarized costs related to treatment with the use of both physical therapies compared are similar and relatively moderate.

## 6. Limitations and Recommendations for Future Studies

The limitations of this study include the lack of information on the therapeutic procedures previously used in the analyzed group of patients, a relatively limited number of patients in the research groups, and a lack of the calculation of sample sizes (as all qualified patients hospitalized in the period of this study have been enrolled in the study), as well as a lack of follow up observations. In order to unequivocally verify the therapeutic effectiveness of the methods assessed, it is essential, as numerous authors emphasize, to conduct multicenter, randomized clinical trials in the future, with the inclusion of a control group, taking more numerous groups of patients into account, in order to define a uniform methodology of procedures (including the dosage of treatments) in case of using individual physical methods.

## 7. Conclusions

Under the experimental conditions, the adopted topical hyperbaric oxygen therapy and topical ozone therapy treatments caused a significant reduction in ulcer area in the treated venous leg ulcers and a reduction in perceived pain ailments, while more effective results were observed for topical hyperbaric oxygen therapy.

## Data Availability

The datasets used and/or analyzed during the current study are available from the corresponding author on reasonable request.

## Abbreviations

ABI	Ankle-brachial index
ACHM	AMERICAN Committee of Hyperbaric Medicine
ADC	Acute distal colitis
ATA	Physical atmosphere
ATP	Adenosine triphosphate
bFGF	Basic fibroblast growth factor
BMI	Body mass index
HBO	Hyperbaric oxygen therapy
NO	Nitric oxide
NOS	Nitric oxide synthase
VAS	Visual analogue scale
VLU	Venous leg ulcers
